# Artificial intelligence as an ally in Brazilian nursing: challenges, opportunities and professional responsibility

**DOI:** 10.1590/0034-7167.2023760301

**Published:** 2023-09-25

**Authors:** Luciano Magalhães Vitorino, Gerson Hiroshi Yoshinari

**Affiliations:** I Faculdade de Medicina de Itajubá. Itajubá, Minas Gerais, Brazil

The current state of nursing in Brazil presents complex challenges and opportunities that require innovative solutions. In this context, artificial intelligence (AI) emerges as a promising tool to address these challenges and seize opportunities. In particular, natural language processing models, such as OpenAI’s ChatGPT, demonstrate the potential to be valuable resources for nurses at all levels of healthcare, highlighting the importance of professional responsibility and the search for balance and safety.

Initially, it is important to emphasize the relevance of the Systematization of Nursing Care (SNC) in providing care based on scientific evidence. In this regard, ChatGPT can play a significant role, as it is capable of analyzing complex clinical data and identifying relevant patterns. Based on this information, it is possible to plan and execute personalized nursing care, resulting in better outcomes for patients^(1-2)^. However, it is essential to emphasize that the responsibility for the outcome of care always lies with the professionals who perform it, not with AI. It is essential to avoid the undue transfer of responsibility for wrong actions by nurses to AI, which, although capable of generating plausible answers, can also present incorrect answers (hallucinations).

AI also plays a relevant role in nursing management, offering support, for instance, in the efficient allocation of human resources, automation of tasks, audit processes and development of Standard Operating Procedures adapted to the needs of the population assisted. By using AI as a support tool, nurses can focus their efforts on the quality of management and clinical care, providing a significant improvement in the efficiency and effectiveness of healthcare services^(3)^. It is essential to consider nurses’ active participation and ethics in technology use to guarantee the safety and quality of the services provided. As already mentioned, there is always a risk of legal issues, misinformation and bias, which users need to be aware of.

Continuing education is a fundamental pillar of nursing professional development, and AI offers personalized and accessible learning opportunities. Through interactive and adaptive platforms such as ChatGPT, nurses can continuously improve their knowledge, skills and competencies. The availability of AI-based educational resources expands the possibilities of professional training, strengthening nursing practice^(2)^.

Moreover, AI can contribute to developing leadership skills and other essential behavioral skills. By providing real-time data on team performance, AI provides valuable input for identifying gaps and opportunities for development. Based on this information, nurses can implement improvement strategies that result in better outcomes for patients and nursing and interdisciplinary teams.

It is worth noting that AI can also play a significant role in nursing research, contributing to increasing the level of scientific evidence. Through the analysis of large volumes of data and the identification of patterns, AI can help Brazilian nurses to develop more robust research with greater impact^(2)^. The proper use of AI as a research support tool can provide significant advances in the area, contributing to the development of evidence-based nursing in Brazil.

However, it is critical to recognize that incorporating AI use in nursing presents challenges that need to be carefully debated and understood. We are still exploring the potential of this tool, and it is crucial to align its use with country-specific guidelines. Responsible adoption of AI requires nurses to validate responses generated by AI based on their clinical knowledge and expertise, ensuring the safety and effectiveness of care.

Undoubtedly, given the possibilities offered by AI, it is essential that Brazilian nursing schools incorporate teaching on AI use in their curricula. Future nurses must be prepared to use this technology ethically and responsibly, understanding its benefits and limitations^(4)^. Teaching about AI should range from understanding the theoretical foundations to the practice of interacting with AI-based systems, including ChatGPT. In this way, nurses will be able to take full advantage of AI’ potential in nursing care.

Such AI models are not restricted to ChatGPT. Med-PaLM 2, a Google tool specifically trained for the health area, with the potential to further increase the effectiveness and safety of nursing care, is already being tested. AIs in general represent cutting-edge technological tools, promising for the improvement of nursing care in Brazil. It is essential, however, to recognize the challenges associated with its implementation and use it as a support tool, not as a substitute for qualified professionals. Only then will we be able to fully exploit the potential of AI and reap the benefits for nursing and, above all, for patients’ well-being.
